# Internet-Based Group Intervention for Ovarian Cancer Survivors: Feasibility and Preliminary Results

**DOI:** 10.2196/cancer.8430

**Published:** 2018-01-15

**Authors:** Ellen M Kinner, Jessica S Armer, Bonnie A McGregor, Jennifer Duffecy, Susan Leighton, Marya E Corden, Janine Gauthier Mullady, Frank J Penedo, Susan K Lutgendorf

**Affiliations:** ^1^ Department of Psychological & Brain Sciences University of Iowa Iowa City, IA United States; ^2^ Orion Center for Integrative Medicine Seattle, WA United States; ^3^ Department of Clinical Psychiatry and Center on Depression and Resilience University of Illinois at Chicago Chicago, IL United States; ^4^ Ovarian Cancer Research Fund Alliance Washington, DC United States; ^5^ Department of Medical Social Sciences Northwestern University Feinberg School of Medicine Chicago, IL United States; ^6^ Life Reset Solutions Chicago, IL United States; ^7^ Division of Gynecologic Oncology Department of Obstetrics & Gynecology University of Iowa Iowa City, IA United States; ^8^ Department of Urology University of Iowa Iowa City, IA United States; ^9^ Holden Comprehensive Cancer Center University of Iowa Iowa City, IA United States

**Keywords:** ovarian cancer, quality of life, feasibility studies, eHealth, psychological stress

## Abstract

**Background:**

Development of psychosocial group interventions for ovarian cancer survivors has been limited. Drawing from elements of cognitive-behavioral stress management (CBSM), mindfulness-based stress reduction (MBSR), and acceptance and commitment therapy (ACT), we developed and conducted preliminary testing of an Internet-based group intervention tailored specifically to meet the needs of ovarian cancer survivors. The Internet-based platform facilitated home delivery of the psychosocial intervention to a group of cancer survivors for whom attending face-to-face programs could be difficult given their physical limitations and the small number of ovarian cancer survivors at any one treatment site.

**Objective:**

The aim of this study was to develop, optimize, and assess the usability, acceptability, feasibility, and preliminary intended effects of an Internet-based group stress management intervention for ovarian cancer survivors delivered via a tablet or laptop.

**Methods:**

In total, 9 ovarian cancer survivors provided feedback during usability testing. Subsequently, 19 survivors participated in 5 waves of field testing of the 10-week group intervention led by 2 psychologists. The group met weekly for 2 hours via an Internet-based videoconference platform. Structured interviews and weekly evaluations were used to elicit feedback on the website and intervention content. Before and after the intervention, measures of mood, quality of life (QOL), perceived stress, sleep, and social support were administered. Paired *t* tests were used to examine changes in psychosocial measures over time.

**Results:**

Usability results indicated that participants (n=9) performed basic tablet functions quickly with no errors and performed website functions easily with a low frequency of errors. In the field trial (n=19), across 5 groups, the 10-week intervention was well attended. Perceived stress (*P*=.03) and ovarian cancer-specific QOL (*P*=.01) both improved significantly during the course of the intervention. Trends toward decreased distress (*P*=.18) and greater physical (*P*=.05) and functional well-being (*P*=.06) were also observed. Qualitative interviews revealed that the most common obstacles participants experienced were technical issues and the time commitment for practicing the techniques taught in the program. Participants reported that the intervention helped them to overcome a sense of isolation and that they appreciated the ability to participate at home.

**Conclusions:**

An Internet-based group intervention tailored specifically for ovarian cancer survivors is highly usable and acceptable with moderate levels of feasibility. Preliminary psychosocial outcomes indicate decreases in perceived stress and improvements in ovarian cancer-specific QOL following the intervention. A randomized clinical trial is needed to demonstrate the efficacy of this promising intervention for ovarian cancer survivors.

## Introduction

Due to difficulties in early detection, the majority of new cases of ovarian cancer (61%) each year in the United States are women diagnosed with advanced-stage cancer [1]. At present, 5-year survival rates for ovarian cancer are relatively low (46% overall) and drop to 29% for those with distant disease and 20% for those over the age of 75 years [1]. Although a significant percentage of patients respond well to initial chemotherapy, treatment efficacy is limited by the development of chemoresistance, and the majority of patients relapse and die from recurrent disease [2]. Thus, ovarian cancer survivors face a unique set of challenges related to late diagnosis, rigorous medical treatments, and poor prognosis.

Given these challenges, it is not surprising that most ovarian cancer survivors report high levels of distress at diagnosis, during treatment, and subsequent disease surveillance [3,4]. During the first year following primary treatment, patients report high levels of anxiety and depressive symptoms, sleep disturbance, fatigue, and treatment side effects (eg, peripheral neuropathy), which impacts their overall quality of life (QOL) [3,5]. Although QOL generally improves over time, patients utilizing avoidant or disengaged coping strategies are at risk for poor QOL [6,7]. Additionally, a greater number of life stressors at 1 year post surgery is associated with poorer concurrent QOL [8]. Numerous studies have identified links between psychological processes and biological pathways related to ovarian cancer progression [9,10]. Specifically, higher levels of perceived social connections with others are related to a more vigorous innate immune response, lower levels of biomarkers related to angiogenesis and invasion, lower levels of the stress hormone norepinephrine in tumor, and genomic changes in tumor indicative of a less aggressive phenotype [10]. Moreover, socially isolated ovarian cancer patients have been shown to have poorer survival [9]. These findings highlight the importance of psychosocial factors in both QOL and survival in ovarian cancer and identify psychosocial factors, particularly emotional social support, as potentially modifiable treatment targets.

A substantial amount of literature has documented the efficacy of psychosocial interventions in improving mood and QOL in cancer patients [11-13]. Although a variety of interventions have been developed for breast cancer survivors [14], development of psychosocial interventions that address ovarian cancer survivorship has been much more limited. Most existing trials have been pilot interventions utilizing small samples [15-20] or exercise- or symptom-based interventions [16,18,21,22]. Barriers to the development and implementation of interventions for ovarian cancer survivors include the relatively small number of ovarian cancer patients at any one treatment site, older age of patients, and physical limitations (eg, neuropathy, fatigue, and cognitive problems), which make it difficult to attend an in-person intervention. A pilot study successfully addressed this difficulty with a 6-month exercise- and phone-based cognitive-behavioral therapy (CBT) intervention [16].

Given the significant problems of distress, social isolation, and poor QOL and the well-established links with biological pathways related to progression, there is a great need for the development of an easily accessible, group-based psychosocial intervention for ovarian cancer survivors. An Internet-based intervention is an ideal approach in this population and offers a number of advantages. First, an Internet-based intervention would provide the opportunity for the small number of ovarian cancer survivors from any treatment center to join with other survivors from around the country. Second, it would enhance accessibility for survivors in rural areas and those with physical limitations who would otherwise not be able to attend an in-person group. Internet-based group interventions have been found to be beneficial for women with breast cancer [23], men with prostate cancer [24,25], and posttreatment cancer survivors [26].

We have developed a 10-week, manualized Internet-based group intervention for ovarian cancer survivors entitled Living WELL: Web Enhanced Lessons for Living for Ovarian Cancer Survivors. The intervention incorporates elements of cognitive-behavioral stress management (CBSM) [27,28], mindfulness-based stress reduction (MBSR) [29,30], and acceptance and commitment therapy (ACT) [31]. It also incorporates topics specifically tailored to address the needs of ovarian cancer survivors such as finding meaning in the face of poor prognosis and managing fear of recurrence. Using a password-secured Web platform that can be used on any Internet-enabled device (eg, tablet, laptop), participants can access a link to the weekly videoconference as well as relaxation and meditation recordings, a journal to record daily gratitude or reflections, and content overviews. The aims of this study were to (1) develop and optimize the intervention and its Web platform and (2) examine the usability, feasibility, acceptability, and preliminary intended effects of an Internet-based group intervention for ovarian cancer survivors.

## Methods

### Study Design

Institutional review board approved all study procedures. Intervention development involved testing the usability, acceptability, and feasibility of the intervention’s content and technological platform (eg, website, tablet, videoconference platform) as well as assessment of intended effects. This testing was done in 3 stages: lab usability testing, field usability testing, and a one-arm field trial of the full 10-week group intervention.

### Recruitment and Participants

Participants were recruited between May 2013 and October 2016. Participants for usability testing were recruited in-person at an oncology clinic at a large Midwestern medical center and by mailings to former participants in a longitudinal ovarian cancer study. Field trial recruitment additionally included listings on the Ovarian Cancer Research Fund Alliance website, flyers in oncology clinics in the Midwestern and Western United States, announcements in local gynecologic oncology newsletters, and referrals from oncology staff or previous participants. Individuals interested in participation called or emailed research staff to receive additional information about the study and participated in a brief eligibility screening. The study was open to English-speaking women with a histological diagnosis of any stage of primary ovarian epithelial cancer, primary peritoneal cancer, or cancer of the fallopian tube following completion of primary chemotherapy. Exclusion criteria included more than one recurrence of ovarian cancer, prior inpatient psychiatric treatment for severe mental illness, or overt signs of severe psychopathology (eg, psychosis) or dementia. Participants who recurred during the field trial and began a new course of chemotherapy (n=2) were able to continue in the intervention; otherwise, participants on chemotherapy were excluded until treatment was completed. All participants provided verbal informed consent.

**Figure 1:**
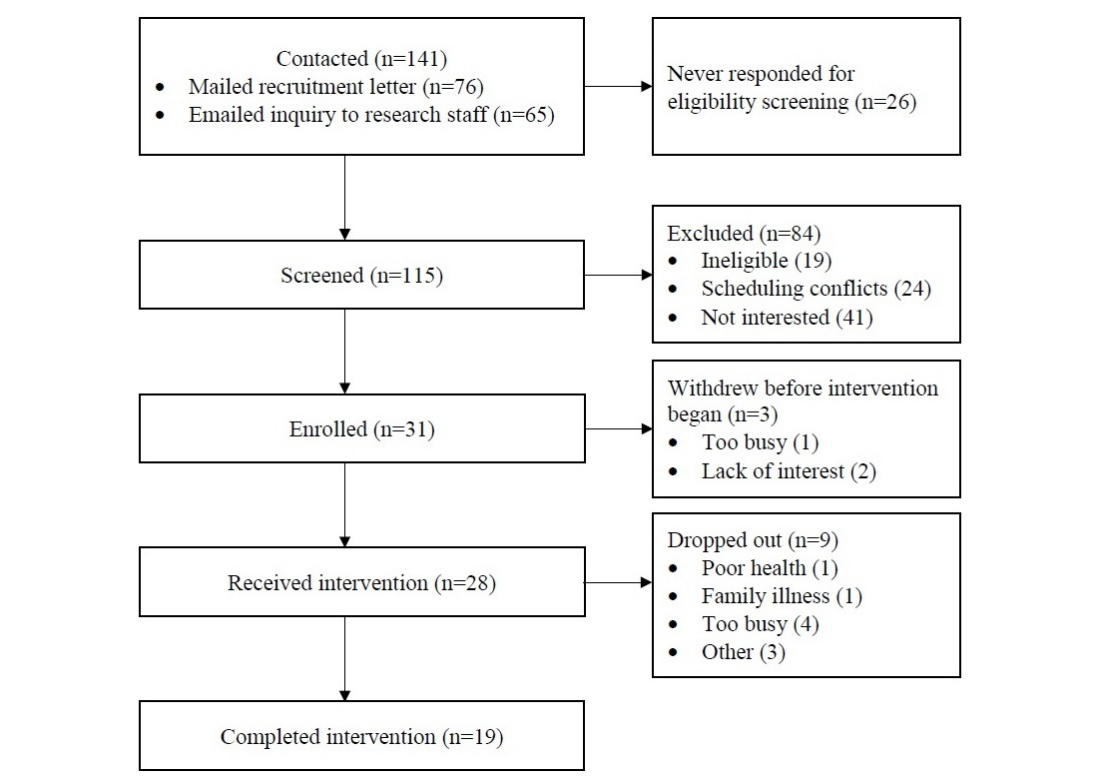
Participant recruitment and retention flowchart for field trial.

For the lab and field usability testing, 25 individuals were mailed a recruitment letter, and 19 individuals were reached by phone to discuss participation. In usability testing, 9 women enrolled and participated (6 lab usability; 5 field usability; 2 participated in both). Reasons for not participating included scheduling conflicts (n=2), being too busy (n=2), and lack of interest (n=6). For the field trials, 76 recruitment letters were sent, in addition to information posting as indicated above. Furthermore, 65 individuals emailed research staff requesting more information. A total of 31 women enrolled in the field trial; 3 withdrew before the intervention began for reasons such as being too busy (n=1) and lack of interest after receiving study materials (n=2). There were 9 participants who attended at least one session but did not complete the intervention; dropouts completed an average of 3 sessions (range 1-7). Reasons for dropout included recurrence with rapid disease progression (n=1), family illness (n=1), being too busy (n=4), and changed mind about participating in a group intervention (n=3). In total, 19 participants completed the intervention (Figure 1).

**Figure 2:**
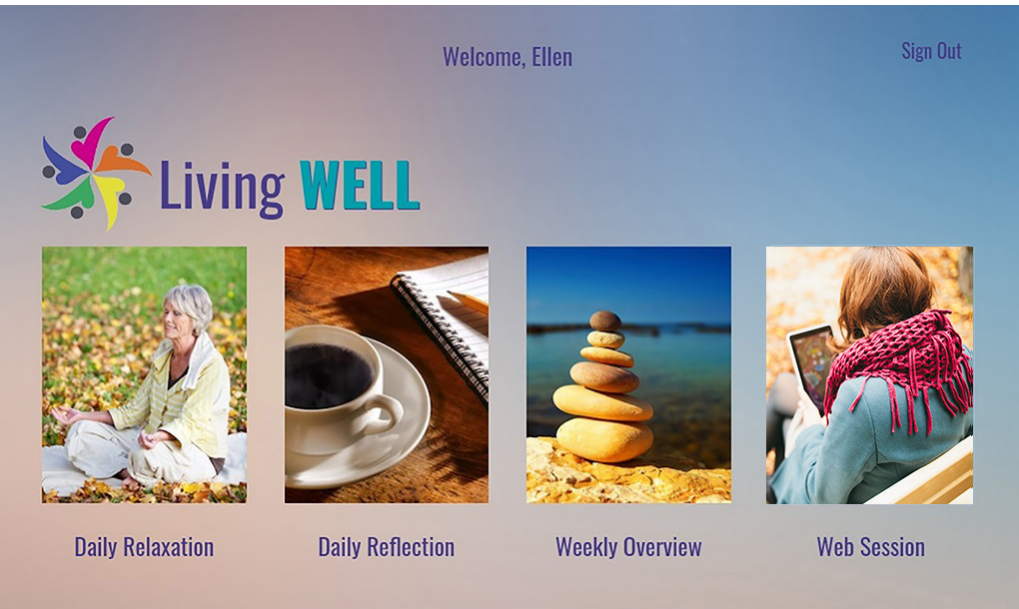
Intervention homepage.

### Web Platform

The Web platform was developed so that participants could easily and securely connect to the group meetings and access intervention content outside of group (see Figure 2 for a screenshot of the Living WELL homepage). It included weekly content overviews, relaxation and meditation recordings, and a journal to record daily gratitude or reflections. It also included a link to the videoconference program, so that participants could connect to the group meetings. WebEx videoconference software (Cisco Systems, Inc., San Jose, CA) was used during the lab and field usability testing and the first 3 field trial groups. In the final 2 field trial groups, Zoom videoconference software (Zoom Video Communications, Inc., San Jose, CA) was used.

Development of the Web platform employed Northwestern University’s (Chicago, IL) Behavioral Intervention Technology Core Facility Extensible Information and Communication Technology Intervention Platform. This platform uses a combination of open-source technologies including Linux, Python, and MySQL server technologies. It is designed for maximum flexibility in interfacing with other technologies while maintaining optimal security and full compliance with the US Health Insurance Portability and Accountability Act (HIPPA). A standardized interface exists to manage user experience as well as enable reports about site usage and data. The intervention’s website was designed and optimized for deployment on tablets but could be accessed with any Internet browser on laptops, desktops, and smartphones.

### Lab and Field Usability Testing

Lab usability testing consisted of a 90-minute session in a quiet room with research staff, in which participants were provided with a Samsung Galaxy 2 tablet as well as oral and written instructions on how to use the device and access the intervention website. Participants were asked to perform a series of tasks involved in the operation of the device and website during which they completed a “think aloud” task where they provide a running commentary while executing tasks. They were also asked to give verbal feedback on obstacles they encountered as well as their overall experience with the technical components of the website.

Field usability testing was done from the participant’s home with study staff available by phone. Upon enrollment, participants were mailed a tablet and user manual. If participants did not have wireless Internet, they received a tablet with 4G capability to provide Internet access. Headphones were supplied if participants needed additional assistance with sound quality. Tablets and headphones were returned upon study completion. In the first session, participants were asked to perform a series of tasks involved in the operation of the device and website. Participants were asked to describe aloud what they were doing to accomplish these tasks. If participants were not yet comfortable with connecting to the videoconference platform on their own, additional practice was offered. Once participants were comfortable connecting to the videoconference, a second and final session was scheduled in which participants were instructed to connect via videoconference with research staff at an appointed time. Before their final session, participants were asked to access the website and review its features at least two other times on their own. In this final session, participants were asked to report their experiences including obstacles with Web activities and evaluation of the website and user manual.

### Field Trial Testing

Enrolled participants were given the option of using their own electronic device or a study-provided tablet. The intervention was a structured 10-week group intervention that met weekly for 1.5 to 2 hour sessions via videoconferencing. Groups were made up of 4-6 ovarian cancer survivors and led by 2 clinical psychologists. In each session, the psychologists introduced a new meditation/relaxation exercise, reviewed homework from the previous week, presented new material, and directed discussion.

**Figure 3:**
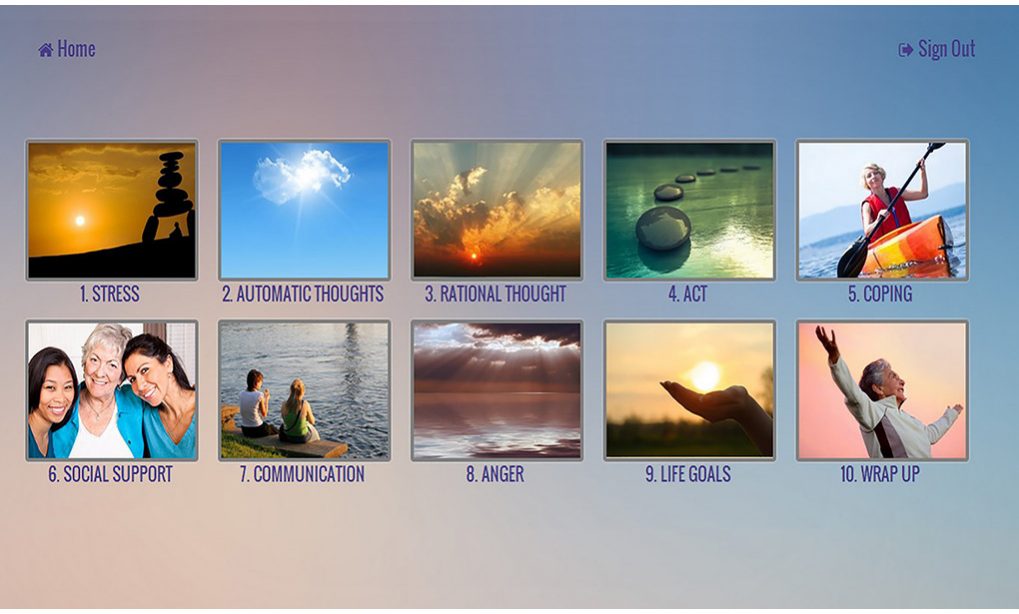
Intervention didactic content, weekly overview.

The intervention was adapted from a CBSM intervention manual in breast cancer [27,28]. Specific CBSM elements in the current intervention included relaxation training and developing stress management skills through in-session didactic material, experiential exercises, and home-based practice. Mindfulness and acceptance-based exercises from MBSR and ACT were incorporated to increase awareness of distressing thoughts and feelings without having to change or avoid them. ACT-based exercises were also used to help identify values and prioritize meaningful activities in one’s life. The intervention consisted of 10 major themes, listed in Table 1, with accompanying relaxation and meditation exercises. A screenshot of the website’s weekly overview page is shown in Figure 3.

Participants were asked to engage in intervention-related activities on their own, including brief homework assignments, daily journaling, and relaxation and meditation exercises. Participants received a printed workbook with the 10-week intervention content and a user manual with instructions on how to navigate the website and videoconference program.

### Evaluation of Lab and Field Usability

All participants completed a survey including demographic and clinical information and ratings of their experience with various technologies (eg, computer, tablet, smartphone). Participants were classified as living in rural or urban location based on county residence using the 2013 US Department of Agriculture Rural-Urban Continuum Codes. For the purposes of this study, codes 7-9 were considered rural locations, a classification method used in previous research in cancer survivors [32,33].

#### Usability

Usability was assessed by completion of common usage tasks, including (1) performing basic tablet functions (eg, turning it on, charging it, entering the proper application); (2) accessing the website (eg, logging in with user id and password); (3) accessing the website features (eg, listening to relaxation recordings); and (4) asking for and utilizing technical support. Research staff recorded the amount of time it took participants to learn and execute tasks as well as their efficiency, frequency, and type of errors made, and ability to perform tasks again after a delay.

Qualitative interviews were used to explore experiences with the system and elicit feedback on the content. Consistent with principles of usability testing, an iterative process was used. Changes in the Web platform were made based on initial feedback. Then, a second group was tested and their feedback was used to further refine the Web interface and content [34,35].

**Table 1:** Weekly session topics and relaxation and meditation exercises.

Week	Session topic	Exercise
1	Introduction: Participants and group leaders introduce themselves. Didactics: Stress response and stress management. Discussion: developing an awareness of personal stressors and finding meaning and sources of personal strength	Deep breathing and progressive muscle relaxation
2	Automatic thoughts: Lecture and exercises to demonstrate the relationships between thoughts, emotions, and physical responses. Demonstration and discussion of appraisal process and cognitive distortions	Passive progressive muscle relaxation and guided imagery
3	Rational thoughts: Lecture and discussion on breaking the vicious cycle of irrational thoughts with rational thought replacement. Demonstration and discussion on alternative responses to negative self-talk	Autogenic relaxation
4	Acceptance: Introduction to mindfulness. Lecture and discussion of avoidance and control strategies and finding effective alternatives through personal values, acceptance, and gratitude	Guided mindful body scan
5	Coping strategies: Lecture and discussion on active problem-focused or emotion-focused coping strategies such as setting prioritizes, asking for help, and relaxation. Exercise on softening in response to painful feelings and emotions	Mindfulness meditation
6	Social support: Lecture on the benefits and types of social support. Exercise on identifying social support and disease-related challenges in communication (eg, fear, changes in intimacy). Discussion of strategies for enhancing support	Mindfulness meditation
7	Effective communication: Lecture on communication styles and effective communication. Exercises and discussion on effective communication and using a mindful moment to become aware of needs and communicate them effectively	Mindfulness meditation
8	Anger: Lecture and discussion on anger. Exercises on identifying patterns of anger expression and steps for dealing with anger such as appraising the situation	Loving kindness meditation
9	Meaning of life: Discussion of how personal values and spirituality help create meaning in life and can change because of cancer. Discussion of strategies to deepen spirituality and meaning such as personal reflection, prayer, and writing	Guided relaxation and visualization
10	Wrap up: Lecture and discussion reviewing material and assessing personal growth. Exercise helping participants develop a stress management maintenance plan	Guided relaxation and visualization

#### Acceptability

Acceptability was measured using a system usability questionnaire (SUQ) modified versions of the System Usability Questionnaire and After-Scenario Questionnaire [36,37] to evaluate user satisfaction and feedback. The questionnaire consisted of rating scales (1 being *strongly agree* to 7 being *strongly disagree*) and free form responses, which asked for general feedback on the overall system (ie, tablet, website, and videoconference platform).

### Evaluation of Field Trial

For all self-report measures in field trial, participants were emailed a link to a Web-based survey tool using Qualtrics (Qualtrics, Provo, UT, USA). These surveys were in accordance with the Checklist for Reporting Results of Internet E-Surveys (CHERRIES; Multimedia Appendix 1) [38]. Structured interviews were conducted over the phone, and the information obtained was used to optimize the intervention manual content (eg, refining language, modifying content and changing the order of presentation, and changing language in relaxation scripts).

#### Acceptability

A 20-item survey, similar to that developed for usability testing, was used to assess acceptability and obtain user satisfaction ratings and feedback. This survey was administered after each group session. It consisted of 10-point Likert scales and free-form responses, requesting feedback on the overall system (eg, website, videoconference platform) and the relevance of that session’s content (eg, topics, relaxation and mediation exercises). Higher scores reflected better ratings. Acceptability and user satisfaction were also assessed with a phone-based structured interview at the conclusion of each 10-week group. Interview questions were separated into themes, including ease of access, usefulness of content, obstacles, and suggestions for improvement.

#### Feasibility

Feasibility was assessed in the field trial testing. It was demonstrated using study recruitment and study retention. Feasibility was also measured by the frequency of 3 intervention-related activities, including number of sessions attended, number of journal entries completed, and meditation/relaxation exercises completed. For each participant, a total for each activity was tallied on the website administrator page by research staff.

#### Preliminary Outcomes

Preliminary psychosocial outcomes of the 10-week intervention were evaluated by self-report measures at baseline and immediately following the last group session.

The Functional Assessment of Cancer Therapy-Ovarian Form (FACT-O) is a 51-item scale measuring QOL in patients with ovarian cancer [39,40]. This scale includes 4 subscales related to general dimensions of well-being, including physical, functional, social, and emotional. It also includes an ovarian cancer-specific subscale with items related to ovarian cancer and treatment-specific QOL issues. The Perceived Stress Scale (PSS) is a 14-item self-report measure used to assess current life stress [41]. Item responses are summed and higher scores indicate more perceived stress.

A total of 2 measures were used to access mood. The Center for Epidemiologic Studies Depression (CESD) scale is a 20-item self-report measure used to assess depressive symptoms [42]. The Profile of Mood States short form is a 37-item inventory, assessing 6 dimensions of mood, including anxiety, depression, anger, vigor, fatigue, and confusion [43]. A total mood disturbance score is calculated from the sum of all scales minus the vigor scale. Both scales are cued to mood over the last week.

The Pittsburgh Sleep Quality Index is a 19-item self-report measure assessing sleep quality and sleep disturbances over a 1-month period [44]. This measure includes 7 subscale scores that are summed to produce a global score with a score. The Social Provisions Scale is a 24-item self-report measure used to assess social support [45]. These subscales are summed to produce a total score.

### Data Analysis

Data from weekly postsession evaluations, website usage, and self-report surveys were downloaded and stored in SPSS 23.0 (SPSS Inc., Chicago, IL, USA). Distributions were examined for normality and outliers. Paired *t* tests were used to examine changes in self-report psychosocial measures from baseline to follow-up. Level of significance was set at *P*<.05. Effect sizes were calculated as the standardized mean differences between the baseline and follow-up time points. The effect sizes and 95% CI reported here were calculated using Hedges’s *g* because this method helps reduce positive bias in small samples [46].

The relationships between intervention-related activities and psychosocial outcomes were examined with those measures that showed statistically significant changes during the intervention. These relationships were examined with bivariate correlations using the number of intervention-related activities (ie, session attendance, journal entries, and relaxation and mediation exercises) and change (delta) scores. Delta scores were calculated by subtracting preintervention scores from postintervention scores to examine changes during the intervention in PSS and ovarian cancer-specific subscale of the FACT-O. A higher score on the PSS indicates greater disturbance; thus, a negative delta score indicates a decrease in perceived stress over time. Higher scores on the ovarian cancer-specific subscale indicate better QOL; thus, a positive delta score indicates improved QOL over time.

## Results

### Participant Characteristics

A total of 6 participants completed lab usability testing and 5 completed field usability testing; 2 of these participants completed both lab and field usability testing. As shown in Table 2, the sample was entirely white, non-Hispanic with an average age of 59.20 years (standard deviation [SD] 14.53). More than half of the sample (56%, 5/9) was college educated. Of the participants, 33% (3/9) lived in rural counties; 89% (8/9) reported an advanced-stage diagnosis (stage III or IV). The average time since diagnosis was 2.5 years (SD 2.12). All participants had completed their primary chemotherapy treatment, although 22% (2/9) were currently receiving additional chemotherapy. Approximately 89% of participants (8/9) had high-speed Internet access and a computer at home. Moreover, 4 participants (44%) reported using videoconference services, and 7 participants (78%) reported using at least one social media service.

In the field trial, 19 participants completed the intervention during 5 successive groups. Group sizes ranged from 3 to 4 completers. As shown in Table 2, the sample was entirely white, non-Hispanic with an average age of 58.89 years (SD 6.87). Participants were located in 7 different states (from New York to Washington), and 4 participants (26%) lived in rural counties. The majority of the sample was college educated (14/19, 74%) and married (14/19, 74%). A total of 9 participants (48%) reported an advanced-stage diagnosis (stage III or IV) with an average time since diagnosis of 2.37 years (SD 1.67). Of these, 3 participants had a disease recurrence and received chemotherapy treatment at some time during the intervention. There were no significant differences in demographic or clinical characteristics between participants who completed the intervention and those who dropped out.

All participants had high-speed Internet access and a computer at home. However, due to poor video or audio quality during group sessions, tablets connected to cellular wireless networks were sent to 2 participants, which improved their connectivity and user experience. A total of 8 participants (42%) used a study-provided tablet to connect to group sessions, whereas 11 (58%) used their own electronic device. Before joining the study, participants reported using a Web-enabled device an average of 4.63 times per week (range 1-6); 6 participants (32%) reported use of videoconference services, and 11 participants (58%) reported use of at least one social media service.

### Usability

There were no significant differences in usability assessments between the lab and field usability testing; therefore, results were combined (Multimedia Appendix 2). Most tasks took less than 10 seconds to learn. Logging into the intervention website (which required participants to type in a username and password) was the one task that took more time. Participants made relatively few errors with 73% (8 of 11 trials) making less than 2 errors. The most common errors were on tasks related to accessing website features for the first time and accessing the videoconference. Notably, most participants were able to correct errors quickly and with little to no instruction from research staff. Additionally, they were more efficient when performing learned tasks and were able to quickly execute tasks even after a delay. Overall, results indicated that participants performed basic tablet functions quickly with no errors, performed Web functions easily with a low frequency of errors, and were able to quickly recover from errors.

### Acceptability

In usability testing, average responses to the SUQ were 1.43, indicating participants were satisfied with the system, felt it was easy to learn and operate, and felt comfortable using it. Themes from qualitative interviews regarding usability were organized into 2 categories: program strengths and program deficiencies.

**Table 2:** Demographic, clinical, and technology use characteristics for usability testing and field trial testing.

Characteristics		Usability testing, n=9	Field trial testing, n=19
Age, mean (SD^a^)		59.20 (14.53)	58.89 (6.87)
White, non-Hispanic, n (%)		9 (100)	19 (100)
Rural residence, n (%)		3 (33)	4 (21)
**Highest level of education, n (%)**			
	High school	4 (44)	5 (26)
	College graduate	5 (56)	7 (37)
	Postgraduate	0 (0)	7 (37)
**Marital status, n (%)**			
	Single	—^b^	3 (16)
	Divorced	—	1 (5)
	Married	—	14 (74)
	Separated	—	1 (5)
**Employment status, n (%)**			
	Full time	1 (11)	4 (21)
	Part time	4 (44)	4 (21)
	Not employed	1 (11)	1 (5)
	Disability	0 (0)	3 (16)
	Retired	3 (33)	7 (37)
**Cancer stage, n (%)**			
	I	1 (11)	5 (26)
	II	0 (0)	5 (26)
	III	7 (89)	8 (42)
	IV	1 (11)	1 (5)
Years since diagnosis, mean (SD^a^)		2.56 (2.24)	2.37 (1.67)
Current chemotherapy, n (%)		2 (22)	3 (16)
**Technology at home (yes), n (%)**			
	Wireless Internet	8 (89)	19 (100)
	Computer	8 (89)	19 (100)
	Tablet	6 (67)	11 (58)
	Smartphone	6 (67)	16 (84)

^a^SD: standard deviation.

^b^Not assessed in usability testing.

Program strengths included the appealing website layout, the ease of program access, and the relevance of information to ovarian cancer survivors. Most participants reported no difficulties navigating the website and were satisfied with its appearance and features, reporting that it was well organized, clear, and inviting. The program deficiency most commonly reported was the need for more of a focus on ovarian cancer survivors in the visual design. After using the system, 8 of the 9 enrolled participants reported that they would be further interested in the intervention. Taken together, these data indicate high levels of usability and acceptability.

Where usability testing revealed issues in the setup of the Web platform, tablet, or instructional materials, feedback was used to make changes in the visual design. These changes included increasing the size of buttons and font for greater readability, using “Teal,” the color associated with ovarian cancer awareness, and extending instructions in the user manual to include the videoconference program.

In field testing, acceptability was examined using the mean responses to questions (see Multimedia Appendix 3) regarding participants’ satisfaction with the session, ability to implement strategies, and comfort with the videoconference platform on a Likert scale from 0 (*not at all*) to 10 (*completely*). The average response to questions on satisfaction with the session and desire to return for the next session were 9.0 (SD 0.74) and 9.20 (SD 0.49), respectively. The average response to questions about session topics and ability to implement strategies discussed was 8.25 (SD 0.81). These results indicate participants were highly satisfied with the group sessions and felt able to implement intervention content. Connectivity issues were the most frequently reported obstacle; however, this varied by group with many participants from the first 3 cohorts reporting difficulties with the videoconference platform. Reported comfort with the videoconference platform increased from an average of 7.76 (SD 1.03) in the first 3 cohorts to 9.08 (SD 0.87) in the last 2 cohorts after the platform switch, indicating less frequent connectivity issues.

Receipt of a physical copy of the participant manual was reported as extremely helpful in promoting ease of access to information, particularly in reviewing past material and practicing concepts discussed in the intervention. Many participants indicated that they referred to the participant manual after the intervention ended to guide them in continued practice of concepts.

Overall, the intervention content was described as useful, well designed, and relevant to ovarian cancer survivors. Participants indicated that drawing examples from the group members’ daily lives was an important aspect of the intervention. In addition, the progression of program material was reported as logical, clear, and relatable. One participant stated:

The progression you did was very helpful. Starting with awareness, then moving on to working with thoughts and tools for coping. It flowed well. Each step built on the ones before. You can’t just say “love yourself,” but by the time we got to self-gratitude, I was ready for it.

Participants reported the intervention had significant impacts on their lives. One participant declared:

This was a real game changer for me

Another stated:

Hands down the best thing I have done for myself since my diagnosis

Finally, many participants reported that a very meaningful aspect of the intervention was the opportunity to connect with other ovarian cancer survivors. In fact, one participant revealed she had:

never met another woman with ovarian cancer before this group

Another indicator of user satisfaction was that participants requested adding additional, monthly group booster sessions after the conclusion of the program to support the connection with other group members and continued practice of concepts learned.

### Feasibility

Of the 96 eligible women screened, 31 enrolled in the study (32% enrollment rate). Scheduling conflicts were a common reason for participant refusal. The retention rate for the field trial was 68% (19 participants completed the intervention out of the 28 who attended at least 1 session), and overall attendance was 88.9% (169/190 sessions) for participants who completed the intervention. The 19 completers attended an average of 8.79 (SD 1.08) group sessions. Notably, participants continued to attend sessions while traveling or on vacation. The average at-home relaxation and meditation practice was 2.78 times per week (range 0.22-7.33), and average journal use was 2.34 times per week (range 0.11-7.89).

### Preliminary Outcomes

Changes in self-reported outcome measures from baseline to follow-up are shown in Table 3. At baseline, average FACT subscale scores were comparable with normative samples of ovarian cancer patients and mixed cancer patients, with higher scores indicating better QOL [39,40]. Following the intervention, there was a nonsignificant trend toward increased total QOL from baseline, 116.22 (SD 16.37), to follow-up, 122.09 (SD 12.52), *t*_17_=1.85, *P*=.08. For FACT-G total scores, a 5-point difference indicates a clinically significant change; for FACT subscales, a 2-point increase indicates clinically significant QOL improvements [47]. Thus, the participant-reported increases related to total QOL scores would be considered clinically significant improvements. A statistically significant increase in ovarian cancer-specific QOL was also observed, with mean scores increasing from 37.11(SD 4.42) to 39.67 (SD 3.56), *t*_17_=2.88, *P*=.01, whereas increases in physical well-being (*P*=.05) and functional well-being (*P*=.06) approached statistical significance.

Significant decreases in levels of perceived stress were reported over the intervention, with mean PSS scores decreasing from 21.28 (SD 7.95) to 18.00 (SD 7.09), *t*_17_=−2.42, *P*=.03. Nonsignificant decreases in depressive symptoms (*P*=.18) and negative mood states (*P*=.17) and increases in social support (*P*=.18) were also reported. No changes in sleep quality were reported.

### Relationships Between Intervention-Related Activities and Outcomes

A final set of analyses examined the associations between the psychosocial outcomes that showed statistically significant changes and intervention-related activities. The number of relaxation exercises completed was significantly correlated with the number of journal entries, *r*=.803, *P*<.001, indicating a strong association between completion of these 2 activities. However, neither of these activities was significantly correlated with the number of sessions attended. The number of relaxation exercises completed was associated with significant decreases in PSS (*r*=−.52, *P*=.03), indicating participants who completed a greater number of relaxation practices reported a decrease in perceived stress over the course of the intervention. Similarly, the number of journal entries completed was also associated with decreases in perceived stress, but this was only marginally significant (*r*=−.45, *P*=.059). There were no significant correlations between any study activities and changes in ovarian cancer-specific QOL during the intervention.

**Table 3:** Changes in psychosocial outcomes from baseline to postintervention (n=18).

Outcome	Preintervention, mean (SD^a^)	Postintervention, mean (SD)	*g* (95% CI)	*P*
FACT^b^: Ovarian cancer QOL^c^	37.11 (4.42)	39.67 (3.56)	0.62 (−0.05 to 1.29)	.01
FACT: Total QOL	116.22 (16.37)	122.09 (12.52)	0.29 (−0.27 to 1.05)	.08
FACT: Physical QOL	22.26 (4.75)	24.18 (2.60)	0.49 (−0.17 to 1.15)	.05
FACT: Social QOL	18.80 (4.34)	17.46 (4.67)	−0.29 (−0.95 to 0.37)	.28
FACT: Emotional QOL	18.00 (3.14)	18.63 (3.63)	0.18 (−0.47 to 0.84)	.31
FACT: Functional QOL	20.06 (4.89)	22.17 (4.05)	0.46 (−0.20 to 1.12)	.06
PSS^d^	21.28 (7.95)	18.00 (7.09)	0.43 (−0.23 to 1.09)	.03
POMS^e^: Negative mood	11.94 (25.18)	5.78 (16.42)	0.28 (−0.37 to 0.94)	.17
CESD^f^: Depression	11.78 (8.95)	9.39 (7.65)	0.28 (−0.38 to 0.94)	.18
PSQI^g^: Sleep quality	7.17 (4.12)	6.78 (3.51)	0.10 (−0.55 to 0.75)	.53
SPS^h^: Social support	84.00 (8.44)	85.78 (8.66)	0.12 (−0.54 to 0.77)	.18

^a^SD: standard deviation.

^b^FACT: Functional Assessment of Cancer Therapy.

^c^QOL: quality of life.

^d^PSS: Percieved Stress Scale.

^e^POMS: Profile of Mood States.

^f^CESD: Center for Epidemiologic Studies Depression.

^g^PSQI: Pittsburgh Sleep Quality Index.

^h^SPS: Social Provisions Scale.

## Discussion

### Principal Findings

This study describes the successful development and preliminary testing of a novel, Web-delivered intervention to address the unique needs of ovarian cancer survivors. The key findings were that an Internet-based intervention for ovarian cancer survivors had high levels of usability and acceptability, moderate feasibility, and preliminary indications suggesting psychosocial effects. In lab and field usability testing, participants were able to operate and navigate tablet and website functions easily with a low frequency of errors and were especially positive about the website’s content and ease of use. These findings demonstrate high levels of usability and acceptability of the intervention’s content and Web platform. Next, in the one-armed field trial of the intervention, participant feedback was especially positive regarding the intervention content and its relevance to ovarian cancer survivors. Preliminary outcome data from the field trial demonstrated statistically significant reductions in perceived stress and improvements in ovarian cancer-specific QOL. Trends toward improved QOL and reduced depressive symptoms were also observed, with medium effect sizes, but did not reach statistical significance.

These preliminary results mirror improvements in QOL and mood from randomized controlled trials of CBSM interventions in women with breast cancer [11,48] and men with early-stage prostate cancer [25]. Notably, the trends toward improved QOL and reduced depressive symptoms in this study align with the preliminary findings of a Web-based CBSM intervention for men with advanced prostate cancer [24]. Adherence to home relaxation practice was relatively low, with participants completing an average of 2.78 at-home practices per week in contrast to the recommendation that participants practice daily. However, this frequency of practice was consistent with levels of home practice reported in similar studies [49]. Notably, participants who used the website’s relaxation and meditation recording feature more often during the intervention reported a decrease in perceived stress after the intervention. This is consistent with findings of similar interventions with human immunodeficiency virus (HIV)-positive men [49], men with prostate cancer [25], women at risk for breast cancer [50], and women with breast cancer [48,51]. Each of these studies reported that the frequency of at-home practice or improvements in perceived ability to relax was associated with psychological and physiological benefits. This highlights the importance of developing approaches to increase at-home practice.

Overall, participants reported positive experiences with the intervention and described it as useful and relevant. Such feedback highlights the advantages of an Internet-based group intervention for ovarian cancer, which provides the opportunity for survivors to connect to one another, transcending the limitations of their treatment setting and physical limitations. Connectivity issues (eg, difficulty hearing or seeing other group members) were commonly reported in early groups, and these were largely addressed by switching videoconferencing platforms. Other obstacles were the time commitment to attending sessions and completing activities outside of the sessions (eg, homework, meditation practices). These obstacles can be addressed in future work by providing participants with an opportunity to set goals for themselves each week. Feedback from participants can help illuminate possible changes that could be made as well as provide group leaders with real-world examples of how participants successfully implement activities into their own lives.

### Limitations

Despite these strengths, it is important to note that the recruitment rate (31 enrolled/96 eligible=32%) for the field trial is a limitation. Although comparable rates are seen in similar research with cancer survivors with advanced disease [22,24,52], participants who completed the intervention may differ from those who chose not to participate or could not be contacted. Notably, scheduling conflicts made up approximately one-third (n=24) of the cited reasons why individuals eligible for the study did not participate. Therefore, providing more flexibility in scheduling group sessions may contribute to improved recruitment rates in the future. Another limitation is the overall retention rate (19 completed/28 attended at least one session=68%) for the field trial. Reasons for participant dropout were disease-related issues or competing priorities, such as family or work, which are similar to the reasons cited for dropout in other interventions with postsurgical ovarian cancer survivors [16,21]. The reasons for dropout underscore the challenges many ovarian cancer survivors face, including noncancer-related stressors and a disease with a high risk of recurrence. Attendance was high among those who completed the intervention, exemplified by attendance even while traveling overseas. Future work should aim to improve retention rates by including clearer expectations during the screening process and providing reminders.

Another limitation is the lack of overall diversity in the sample, which limits generalizability of the study. The sample of completers was entirely white, non-Hispanic women and most were college-educated. Despite these limitations in generalizability to all ovarian cancer survivors, this sample included a few participants who lived in rural areas. Therefore, the results of this study lend some insight into the impact that interventions can have on survivors from rural locations. A fourth limitation is the small sample size in the field trial that limited power of statistical analyses. Finally, without a control group, factors other than the intervention could have influenced the preliminary outcome results. Therefore, future work will need to include a randomized controlled trial with an active control group as well as longitudinal follow-up after the intervention.

### Conclusions

Our findings suggest that an Internet-based group intervention is highly usable and acceptable for ovarian cancer survivors with moderate levels of feasibility at this time. Preliminary data suggest decreases in perceived stress and improvements in QOL, following the intervention. An Internet-based group may be especially well suited for this population, given the small number of ovarian cancer patients at any one treatment site. Future research with this intervention should focus on a randomized controlled trial to evaluate its efficacy on clinically relevant cancer outcomes such as mood and QOL. Other areas of future research include determining at which points in the survivorship trajectory an intervention such as this is most helpful, as well as assessing the potential effects on outcomes over time and examining potential effects on biological mediators that are known to modulate cancer growth.

## Appendix

Multimedia Appendix 1 Checklist for Reporting Results of Internet E-Surveys.

Multimedia Appendix 2 Results of the usability assessment results for laboratory and field usability.

Multimedia Appendix 3 Weekly session evaluation scores averaged across 10 weeks.

## Abbreviations

ACT: acceptance and commitment therapy
CBSM: cognitive-behavioral stress management
CBT: cognitive-behavioral therapy
CESD: Center for Epidemiologic Studies Depression
CHERRIES: Checklist for Reporting Results of Internet E-Surveys
FACT-O: Functional Assessment of Cancer Therapy—Ovarian Form
MBSR: mindfulness-based stress reduction
QOL: quality of life
PSS: perceived stress scale
SUQ: system usability questionnaire
