# Supplementary material for: Internet-Based Group Intervention for Ovarian Cancer Survivors: Feasibility and Preliminary Results
Source: JMIR Cancer. 2018 Jan 15;4(1):e1. doi: 10.2196/cancer.8430 (PMC5789163; doi:10.2196/cancer.8430)
Supplement: Multimedia Appendix 1 [file cancer_v4i1e1_app1.pdf]

Multimedia Appendix 1

Checklist for Reporting Results of Internet E-Surveys

Design
The e-surveys for the present study were to collect data from field trial participants, and two types of e-surveys were administered. The first was the pre/post-intervention survey, which was administered before the first group session (pre-intervention) and after the final group session (post-intervention). The pre/post-intervention surveys contained the psychosocial measures to assess preliminary intervention outcomes. The pre-intervention survey also contained items asking about relevant demographic and clinical information. The other e-survey was a session evaluation survey, which was administered following each group session.

IRB Approval and Informed Consent Process
The study and all surveys were approved by the institutional review board. The informed consent process was conducted over the phone. An enrollment letter was also mailed to participants, which outlined the elements of informed consent reviewed over the phone. Data was stored appropriately following institutional guidelines.

Development and Pre-testing
The session evaluation survey was based on the survey used in usability testing. The measures in the pre/post-intervention survey was based on intended intervention targets (e.g. quality of life). Both surveys were pre-tested for usability and technical functionality on different internet-enabled devices by the research team.

Recruitment Process
All surveys were closed to those enrolled in the study. Potentially eligible individuals could contact research staff by email for additional information about the study. Following initial contact, if individuals were still interested in participation they completed a brief eligibility screen over the phone. No advertising was used to promote the surveys.

Survey administration
All the surveys were administered by email to participants using Qualtrics (Qualtrics, Provo, UT, USA), a web-based survey tool. All surveys were voluntary and items left blank were coded as missing. No incentives were offered to complete the surveys.

The pre-intervention survey was 12 pages and had between 3 and 37 items per page. It was open for two weeks before the first group session The post-intervention survey was 8 pages and had between 5 and 47 items per page. It was open for two weeks immediately after the last group session. The weekly session evaluation was a page and had 20 items. It was available immediately after each group session for one week.

A completeness check was not enabled on any of the surveys. Participants had the option to go back and review or change responses. Items were not randomized or alternated. Adaptive questioning was not used.

Response Rate
The completion rate for pre-intervention survey was 93% (26 surveys started/28 survey emails sent). The completion rate for post-intervention survey was 100% (19 surveys completed/19 survey emails sent). The completion rate for the session evaluation survey was 81% (150 surveys completed/185 survey emails sent).

Preventing Multiple Entries
All surveys were close and did not involve a login because each participant was sent their own link to the surveys. Therefore, a survey was not displayed a second time once the participant had complete it.

Analysis
Data from the pre/post-intervention surveys was analyzed for the 19 participants that completed the intervention. Data from the session evaluation survey was analyzed for all available data. Time-stamps were recorded, but was not used as a cut-off point to exclude rapidly submitted surveys. Instead, time-stamps were used with the session evaluation survey to assist in matching session evaluation surveys to the appropriate time points. No statistical correction for missing values was used.
