# Supplementary material for: Internet-Based Group Intervention for Ovarian Cancer Survivors: Feasibility and Preliminary Results
Source: JMIR Cancer. 2018 Jan 15;4(1):e1. doi: 10.2196/cancer.8430 (PMC5789163; doi:10.2196/cancer.8430)
Supplement: Multimedia Appendix 2 [file cancer_v4i1e1_app2.pdf]

Multimedia Appendix 2

Results of the usability assessment results for laboratory and field usability (n = 11 trials[a]).

Task	Function	Seconds to perform<br>Mean (range)	Errors<br>Total (range)
Log into website			
	Learnability[b]	103.7 (0.5-720.0)	2 (0-1)
Access daily gratitude journal			
	Learnability	8.8 (0.5-60.0)	4 (0-2)
	Memorability[c]	1.0 (0.5-4.0)	1 (0-1)
Access relaxation recordings			
	Learnability	1.2 (0.5-8.0)	4 (0-1)
Access weekly overview			
	Learnability	3.7 (0.5-15.0)	3 (0-1)
	Memorability	2.1 (0.5-10.0)	2 (0-1)
Access weekly overview independently			
	Efficiency[d]	0.8 (0.5-3.0)	2 (0-1)
Access web conference			
	Learnability	2.2 (0.5-10.0)	5 (0-2)
Turn off tablet			
	Learnability	0.5 (0.5-0.5)	0 (0)

[a]The 11 trials include six usability testing trials and the five field usability testing trials. These trials included nine participants as two participants completed both lab and field usability testing.
[b]Learnability was assessed by the number of seconds to first perform a new task.
[c]Memorability was assessed by the number of second to perform a task after a delay. It was only meaningful in the two specific contexts reported above.
[d]Efficiency was assessed by the number seconds to perform a leaned task. It was only meaningful in the specific context reported above.
