# Supplementary material for: Internet-Based Group Intervention for Ovarian Cancer Survivors: Feasibility and Preliminary Results
Source: JMIR Cancer. 2018 Jan 15;4(1):e1. doi: 10.2196/cancer.8430 (PMC5789163; doi:10.2196/cancer.8430)
Supplement: Multimedia Appendix 3 [file cancer_v4i1e1_app3.pdf]

Multimedia Appendix 3

Weekly session evaluation scores averaged across 10 weeks.

Question	Mean (SD)
What is your satisfaction with today's group session?	9.00 (0.74)
What is your desire to return to the next group session?	9.20 (0.49)
To what extent did today's discussion adequately address the topics?	8.75 (0.64)
To what extent do you feel able to implement the strategies discussed in today's session?	8.25 (0.81)
How comfortable were you with the video conferencing platform part of the meeting today?	8.26 (0.90)[a]
	7.76 (1.03)[b]
	9.08 (0.87)[c]

Higher scores reflect better ratings. The scale ranges from 0 to 10.
[a]The average score for all field trial groups
[b]The average score for field trial groups 1-3
[c]The average score for field trial groups 4-5
